# Range expansion of two Triatominae species in Oaxaca, Mexico: first report and molecular identification of *Triatoma barberi* and *Triatoma dimidiata* in the Cañada region

**DOI:** 10.1590/0074-02760260093

**Published:** 2026-10-16

**Authors:** Benjamín de Israel Carbajal-Olivier, Laura Isela Cruz-López, Saúl González-Guzmán, Rocío Rosas-López, Vladimir Paredes-Cervantes, Edward T Bagu, Citlalli Regalado-Santiago, Araceli Vaquero-Vera, Patricia González-Cano

**Affiliations:** 1 Universidad de La Cañada, Departamento de Farmacobiología, Teotitlán de Flores Magón, Oaxaca, México; 2 Hospital Regional de Alta Especialidad de Zumpango, Unidad de Enseñanza e Investigación, Estado de México, México; 3 Centro Médico Nacional La Raza, Unidad de Investigación Médica en Inmunología e Infectología IMSS, Ciudad de México, México; 4 University of South Dakota, Sanford School of Medicine, Vermillion, South Dakota, USA

**Keywords:** Chagas disease, vector, range expansion, epidemiology

## Abstract

**BACKGROUND:**

Chagas disease (CD) is a chronic, disabling vector-borne disease caused by *Trypanosoma cruzi* and transmitted to humans by infected triatomine insects during blood meals. CD is currently endemic in 21 Latin American countries and the United States of America, with factors such as climate change, urbanisation, and migration contributing to the expansion of the vector’s range from endemic to non-endemic regions. Globally, 44 countries have reported CD cases, indicating that it is an important public health problem.

**OBJECTIVES:**

To document the presence and geographic distribution of *Triatoma dimidiata* and *Triatoma barberi* in previously unreported areas, and to assess their epidemiological relevance for CD.

**METHODS:**

Molecular, entomological, and spot pattern analyses were performed. DNA was extracted from triatomines, the ITS2 region amplified, cloned, and sequenced. An extensive search for reports on triatomine in Oaxaca was conducted from 2019 to 2024, and a distribution map was generated.

**FINDINGS:**

We report the range expansion of two species to communities in La Cañada region. The CD vector range expansion poses an increased risk of infection in newly affected communities, given the higher prevalence of CD in Oaxaca.

**MAIN CONCLUSIONS:**

Awareness of triatomine distribution is essential to prevent the spread of CD into non-endemic areas and to identify emerging endemic zones for epidemiological surveillance and disease control.

Chagas disease (CD) is a vector-borne parasitic disease caused by the protozoan parasite *Trypanosoma cruzi*.^1^ It is predominantly transmitted by an infected triatomine vector, although transmissions may occur through congenital transmission, perinatal exposure, blood transfusion, organ transplantation, and oral ingestion of contaminated foods or beverages.^2^ Less common, additional transmission routes, such as laboratory accidents, have been reported.^1^ CD is currently listed by the World Health Organization (WHO) as a neglected tropical disease; however, it remains a public health concern worldwide.^3^ Recent estimates indicate that approximately 6-7 million people are infected with *T. cruzi* worldwide, highlighting CD’s continued public health importance.^4^ ^5^ As of December 2024, cases were reported in 44 countries in addition to the 21 Latin American countries. CD cases have also been documented in the United States of America,^6^ Canada, and 17 European countries.^7^ Nevertheless, the majority of cases occur in Latin America, where approximately 1.05% of the population is estimated to be affected.^4^ ^5^

In Mexico, CD is reported across all 32 states.^8^ A previous study estimated a national seroprevalence of 3.38%.^9^ According to the Mexican Epidemiology Bulletin, by the end of 2025, a cumulative total of 37 acute and 587 chronic CD cases had been reported nationwide. During the same period, Oaxaca state reported five acute and 73 chronic cases, corresponding to an incidence of 12.43%.^10^ ^11^ WHO has ranked Mexico as the third most affected country for CD and the second highest worldwide for vector-borne transmission.^12^ In endemic areas, CD is primarily transmitted to humans and animals by the hemipteran insect of the subfamily Triatominae.^13^ The subfamily Triatominae comprises 154 extant species grouped into 18 genera,^14^ and all hematophagous species are capable of transmitting *T. cruzi*.^13^ Thirty-one of the 154 species have been identified in Mexico,^15^ of which eight have been found in Oaxaca state, in the southwest of Mexico.^16^

The impact of the triatomine vector on the transmission of CD was demonstrated by the Southern Cone Initiative, which showed that controlling the vector population led to a 65% reduction in CD infections.^17^ The transmission of *T. cruzi* depends not only on the type, abundance, distribution, and feeding habits of vector species in domestic, peridomestic, or sylvatic habitats,^18^ ^19^ but also on host availability and environmental conditions. Some *Triatomine* species feed on specific hosts according to their presence in domestic or peridomestic settings, whereas others feed opportunistically on mammals in sylvatic environments.^6^ The presence of an infected vertebrate, either human or domesticated animals, is crucial not only for infecting a triatomine vector,^19^ but also for sustaining the transmission cycle of CD.^20^ Therefore, monitoring the co-distribution of infected vectors and hosts in endemic areas is essential for preventing disease transmission.^21^ Additionally, accurate identification of triatomine species and evaluation of their vectorial competence are epidemiologically important for the control of CD within affected communities.^22^

Due to limitations in surveillance systems, diagnostic challenges, and unequal access to healthcare, CD is likely underreported in Mexico, particularly in high-burden states such as Oaxaca. This underreporting may contribute to substantial discrepancies between officially reported cases and epidemiological estimates.^11^ Field-based studies conducted in southern Mexico support this concern, as serological screening in communities, such as Pochutla, Oaxaca, has revealed a high prevalence of infection within the examined population.^23^ Previous studies in Mexico have also shown that the presence of infected triatomine vectors in and around human dwellings is strongly associated with increased entomological risk for *T. cruzi* transmission.^24^

In this context, the documented occurrence of triatomine vectors highlights the potential for ongoing, yet undetected, transmission. From a One Health perspective, interaction among human populations, triatomine vectors, animal hosts, and environmental conditions is central to understanding the transmission dynamics of *T. cruzi* in endemic regions. Therefore, the aim of this study was to evaluate the presence and distribution of triatomine vectors in rural communities of the Teotitlán district, Oaxaca, in order to assess their potential role in local transmission risk. We hypothesised that the presence of triatomine vectors in and around human dwellings is associated with increased potential transmission risk in the study area.

## MATERIALS AND METHODS

*Triatomine distribution in Oaxaca, Mexico* - A data search for triatomine distribution in Mexico was performed using the terms “Chagas_disease_Oaxaca,” “Chagas_disease_Vector_Oaxaca,” and “Triatomine_Oaxaca” in the PubMed and SciELO databases. The Global Biodiversity Information Facility (GBIF) database^25^ was also used to find triatomine reports in Mexico. The GBIF search was limited to the geographical coordinates, limiting Oaxaca state; N: 18.71º N; S: 15.60º N; W: 98.65º W, and E: 93.70º W. These searches provided the municipality names where *Triatoma dimidiata* and *Triatoma barberi* were previously identified.

*Triatomine collection* - Triatomine specimens were obtained through community participation during a serological survey conducted in the La Cañada region of Teotitlán de Flores Magón, Oaxaca. Educational outreach activities were carried out to inform local populations about CD and its vectors. Following these sessions, community members voluntarily provided triatomine specimens encountered in domestic and peridomestic environments. Community-based educational activities and active case-finding efforts facilitated the detection of triatomine vectors in municipalities where they had not previously been reported.

Adult and nymphal *T. barberi* vectors were collected from both intradomicile and peridomicile habitats within the municipality of Teotitlán de Flores Magón (TFM). In Mazatlán Villa de Flores (MVF), homeowners captured *T. dimidiata* specimens inside their homes and submitted them to the research team. As a result, specimen collection was opportunistic and did not follow standardised entomological sampling protocols. All the collected samples were placed in labelled plastic containers and transported to the Universidad de la Cañada for analysis.

*Triatomine geographical distribution in Oaxaca’s map* - All municipalities in Oaxaca previously reported to have triatomines were compiled into a table, and their geographical locations were mapped. Species locations were colour-coded according to the order of appearance in the search results, and a dot was added to municipalities where more than one species was reported.

*Triatoma barberi Usinger (1939) and T. dimidiata Latreille (1811) morphometric identification* - *T. barberi* and *T. dimidiata* were morphologically identified using the key features published by Lent and Wygodzinsky.^26^ The diagnostic features used were body size, colouration, head proportions, pronotum, and abdomen width, as well as the morphology of the clypeus, antennae, and femora.

*Triatoma dimidiata (Latreille 1811) quantitative imagerie identification* - The *T. dimidiata* haplogroup was identified using the image-based dorsal spot pattern analysis previously described.^27^ Briefly, digital images of the specimens were taken and processed. The head and legs were manually cropped from the captured images, and the background colour was removed using Photo Editor 3.0. The resulting image was converted to black-and-white to acquire the spot pattern using ImageJ software^22^ with the provided macro.^28^ The final processed image was then superimposed on the previously generated heat map for haplogroup identification.^27^

*Molecular identification of the Triatome vectors, T. dimidiata and T. barberi*: *DNA extraction from adult and nymph triatomines* - DNA from adult triatomine was extracted by injecting 500 µL of lysis buffer (50 mM Tris, 300 mM NaCl, 0.1% SDS, 0.02% NaN3, and 10% glycerol; pH 8.0) through the rectum; the tissue was removed, disrupting the abdominal cavity with a 21G sterile needle attached to the syringe. DNA from nymph triatomine was isolated following maceration of the entire nymph in a tissue grinder (Wheaton, NJ, USA.) with 1000 µL of lysis buffer. The lysate was transferred to an eppendorf tube containing 100 µg/mL of recombinant proteinase K (Invitrogen). The samples were mixed and incubated in a Thermomixer comfort (Eppendorf, Germany) for 2 h at 55ºC with shaking at 10 min intervals, and DNA was extracted using the neutralised phenol-chloroform protocol.^29^

*Polymerase chain reaction (PCR) primers* - Specific primers previously reported for the Internal Transcribed Spacer-2 region (ITS-2) from *T. dimidiata*, ITS2_200F (5’TCGYATCTAGGCATTGTCTG3’) and ITS2_200R (5’CTCGCAGCTACTAAGGGAATCC 3’)^30^ were used to amplify the ITS-2 of *T. dimidiata* captured in MVF. A primer set for *T. barberi* ITS2 was designed using NCBI Primer-BLAST^31^ to identify the Triatome species collected from TFM; BITS-2F (5’ ATCTAGGCAATGTCTGGGCG 3’) and BITS-2R (5’ GCATTGCAGTTTGATACCGACA 3’) primers to detect *T. barberi*.

The endpoint PCR reactions were performed using a T100 thermal cycler (BioRad, CA, USA) with the following conditions: *T. dimidiata* were incubated at 95ºC for 2 min followed by 35 cycles at 95ºC for 30 s, 62ºC for 30 s, and 72ºC for 30 s, and a final extension at 72ºC for 1 min as previously reported.^30^ *T. barberi* PCR was performed as follows, 95ºC for 2 min then 35 cycles at 95ºC for 30 s, 60ºC for 30 s and 72ºC for 30 s, and a final extension at 72ºC for 1 min.

*Sequencing* - The PCR products were sequenced at the Molecular Biology Unit, Cell Physiology Institute, Mexican National Autonomous University. Amplicon sequences obtained were compared against the BLAST (Basic Local Alignment Search Tool) database for *T. dimidiata* and *T. barberi* to confirm the identity of collected samples. The Tree Viewer 1.19.1 tool was used to identify the *T. barberi* clade.^32^

*Ethics* - The present work was approved by the ethics committee at La Cañada University.

## RESULTS

*Triatomine species distribution per municipalities in Oaxaca* - The database search revealed that, prior to our study, eight triatomine species had been identified in 150 of the 570 municipalities (M) in Oaxaca, Mexico [Fig. 1A, Supplementary data (Table I), municipality key and name].^16^ ^25^ ^33-37^ With the addition of our findings, triatomine species have now been documented in 151 municipalities. *T. barberi* was the most widely distributed in 80 municipalities (M),^16^ ^25^ ^38^ followed by *T. dimidiata* in 55 (M),^16^ ^25^ ^34^ *Triatoma mazzottii* in 34 (M), *Triatoma phyllosoma* in 36 (M),^16^ ^25^ *Triatoma pallidipennis* in 15 (M), *Triatoma bolivari* in seven (M), *Triatoma nitida* in three (M), and *Rhodnius prolixus* was recorded in two of the 151 (M) reported in Oaxaca state.^25^ However, the latter species is now considered eradicated from Mexico, as no confirmed occurrences have been reported since 2010^39^ [Supplementary data (Table II), (triatomine distribution per municipality)].

**Fig. 1:**
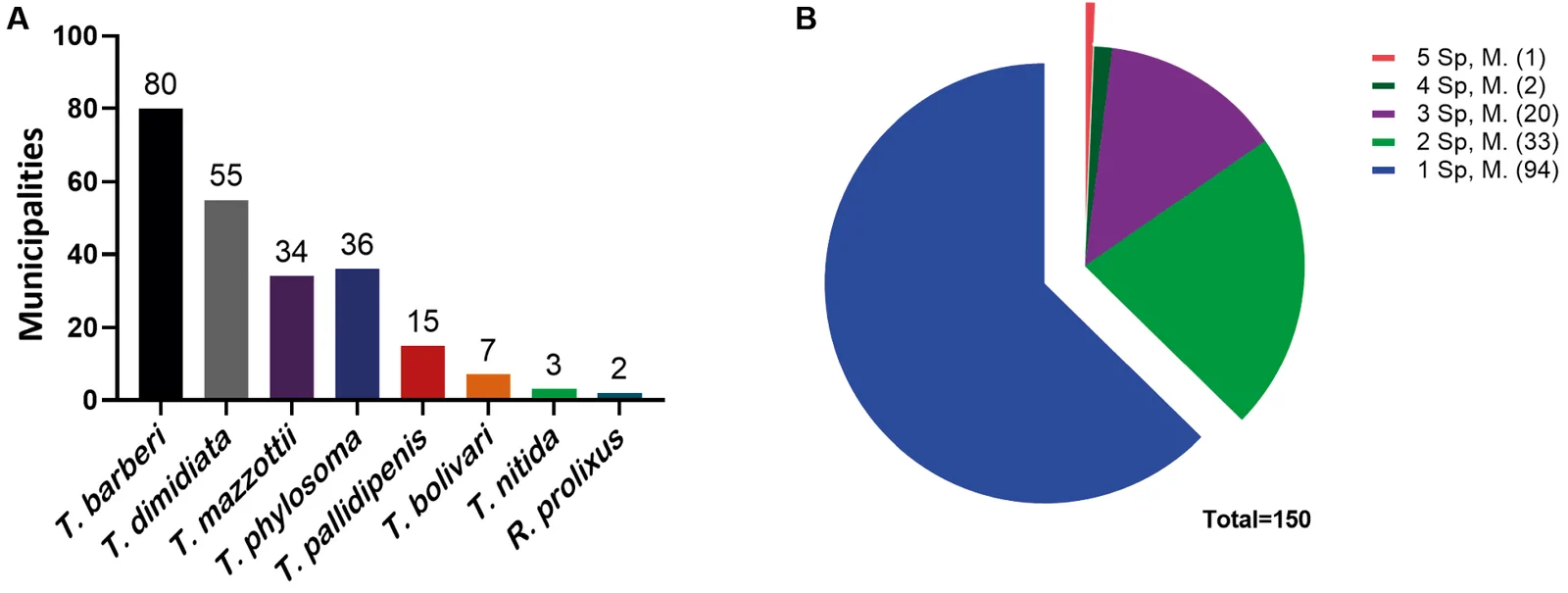
distribution and co-occurrence of Triatomine species (sp.) in Oaxaca, México. (A) The most widely distributed Triatomine species was *Triatoma barberi* in 80 municipalities (M); *Triatoma dimidiata* in 55; *Triatoma mazzottii* in 34 M; *Triatoma phylosoma* in 36 M; *Triatoma pallidipenis* in 15 M; *Triatoma bolivari* in 7 M; *Triatoma nitida* in 3 M and *Rhodnius prolixus* in 2 M.( B) Co-occurrence of Triatomine sp.; five sp. were found in 1 M, two sp. in 2M, three sp. in 20 M; two sp. in 33 M, and one sp. in 94 M.

*Co-localisation of triatomine species in Oaxaca’s municipalities* - Nejapa de Madero was the most infested municipality, with five species co-localised: *T. barberi*, *T. dimidiata*, *T. mazzottii*, *T. phylosoma*, and *R. prolixus*. Santa María Colotepec and Santa María Tonameca ranked second, each with four species co-localised: *T. dimidiata*, *T. mazzottii*, *T. bolivari* and *T. phylosoma*. Across Oaxaca, three species were co-localised in 20 municipalities, two species in 33 municipalities, and one species in 94 of 151 municipalities [Supplementary data (Table II), Figure (B)].

*First records of T. barberi and T. dimidiata in the Teotitlán District* - Here, we report, for the first time, the presence of *T. barberi* and *T. dimidiata* in TFM (municipality #545) and MVF (municipality #58), respectively, in both municipalities of Teotitlán District. These findings increase the known distribution of *T. barberi* and *T. dimidiata* to 80 and 55 municipalities in Oaxaca, respectively (Fig. 2).

**Fig. 2:**
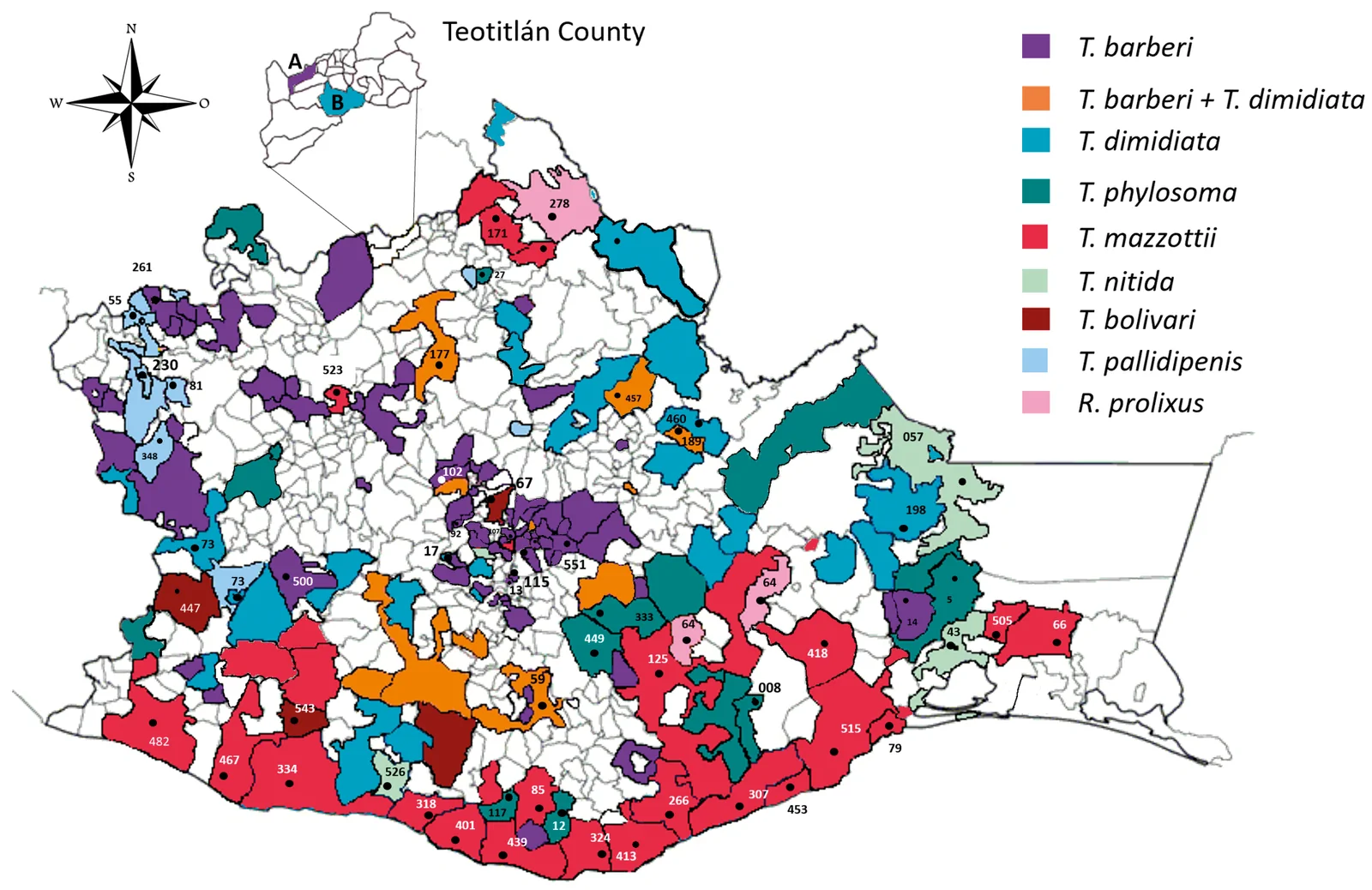
the distribution of Triatominae species (sp.) in Oaxaca, Mexico. Eight Triatominae sp. were recorded in 151 of Oaxaca’s 570 municipalities. The map shows the number of sp. in each municipality using a colour code. Newly reported occurrences of *Triatoma barberi* and *Triatoma dimidiata* are highlighted in the inset, showing Teotitlán de Flores Magón [TFM (A)] and Mazatlán Villa de Flores [MVF (B)].

Four *T. barberi* adult females were captured in a peridomestic habitat at the following coordinates: 18º08’04.0”N 97º05’45.9”W, 18º08’08.9”N 97º05’52.6”W, and 18º08’03.2”N 97º05’53.9”W. One of the four females had copulated and subsequently laid eggs the following day. Additionally, one adult and four nymphs were captured indoors, inside a closet of a house with flat walls and a concrete ceiling, located at 18º08’14.4”N 97º04’56.8”W.

Coordinates were recorded in situ using handheld GPS devices and are presented solely for geographic reference; no spatial statistical analyses were performed. All collection points were located in TFM at 1,020 metres above sea level (masl), at the base of the Sierra Mazateca hillside.^40^ The weather in this region is mild to dry, with light rain from June to October. The annual mean temperature and precipitation are approximately 21ºC and 1,010 mm, respectively. Vegetation is predominantly tropical deciduous forest and “cuajiotal”, characterised by unbranched trees with photosynthetic stems. The ecosystem is a low, deciduous, jungle-like environment on the hillside where *T. barberi* was captured.

*Triatoma dimidiata* was captured indoors in a house in MVF (18º01’59.3”N 96º54’52.3” W). The municipality is primarily covered by pine-oak forest wooded area at 1,140 masl, with four tree species: *Pinus teocote*, *Quercus* sp., *Populus alba*, and *Pinus sylvestris*. The climate is mild to cold, with temperatures ranging from 12ºC to 23ºC, and heavy rain from June to October, with precipitation ranging from 1000 mm to over 3000 mm.^41^ The house where the specimen was collected was surrounded by wooded vegetation and domestic animals, including pigs, dogs, cats, and chickens. Indoors, the specimen was found in a room with a concrete ceiling and mortar stone walls containing crevices in the mortar joints.

*Morphological identification of T. barberi Usinger, 1939* - Adult *T. barberi* (Fig. 3A) specimens measured 1.9 to 2.0 cm in length (head to abdomen). The anteocular region was approximately twice as long as the postocular region. The head was convex, and the connexivum markings ranged from orange to reddish. The first antennal segment was approximately two-thirds the length of the clypeus apex. All specimens had black eyes and a black pronotum with two submedian carinae. A groove was present in the central depression of the scutellum, extending from the base to the tip of the scutellar process, consistent with the previously published description.^26^

**Fig. 3:**
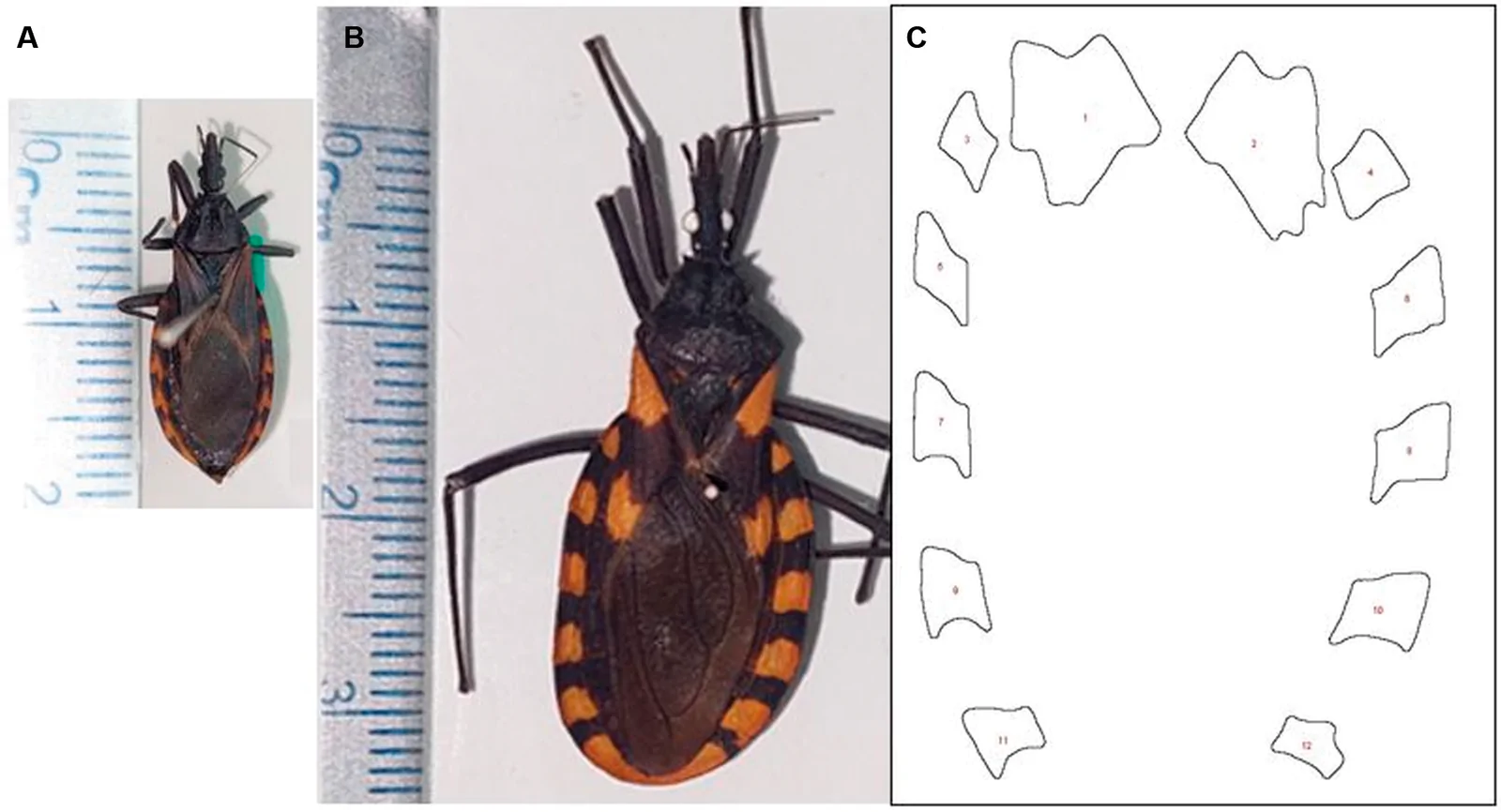
*Triatoma barberi* and *Triatoma dimidiata* specimens. Both species (sp.) had a size consistent with previous reports.^22^ ^53^ (A) Dorsal view of *T. barberi* female captured in Teotitlán de Flores Magón (TFM). (B) Dorsal view of *T. dimidiata* male captures in Mazatlán Villa de Flores (MVF). (C) ImageJ Spot pattern from *T. dimidiata* male (2B) corresponding to *T. dimidiata* H2 haplogroup.^27^

*Morphological identification of T. dimidiata Latreille 1811* - The male *T. dimidiata* specimen (Fig. 3B) measured 3.3 cm in length. The corium was orange-yellow with large central dark spots; the orange-yellow areas were half the size of the connexival segments. The anteocular region was about three times longer than the postocular region. Both the eyes and ocelli were black. The first antennal segment reached the level of the clypeus apex, consistent with previous descriptions.^26^ ^42^

*Triatoma dimidiata haplogroup identification* - The dorsal spot pattern of *T. dimidiata* was analysed using an ImageJ automated identification process (Fig. 3C). The resulting pattern was qualitatively compared with the previously reported heat map for haplogroups 1 to 3. The analysis indicated that the *T. dimidiata* specimen from MVF belongs to haplogroup 2.^27^

*Molecular identification* - PCR was performed as described in the materials and methods; amplicons of the expected sizes of 115 bp for *T. barberi* and 320 bp for *T. dimidiata* were obtained (data not shown). The resulting sequences were submitted to the GenBank, and BLASTn analysis confirmed the species identity.

For *T. dimidiata*, BLASTn analysis showed E values ranging from 7e-107 to 2e-88, with 93.98% to 90% identity. Query coverage reached 98%, while subject (GenBank reference) coverage was 91%, reflecting the shorter length of our sequence relative to the reference sequences.

Only two ITS-2 sequences were available for *T. barberi*. Our query sequence showed 100% identity and 100% coverage with both sequences (accession numbers JX872261.1 and AJ293590.1), with E-values of 6e-26.

In contrast, comparisons with *Triatoma protracta* sequences showed lower similarity despite complete coverage: 92.75% identity (E value = 1e-17; accession numbers JX872263.1 and JQ282713.1), and 91.43% identity (E value = 6e-16; accession numbers JQ282715.1 and JQ282714.1).

**Fig. 4:**
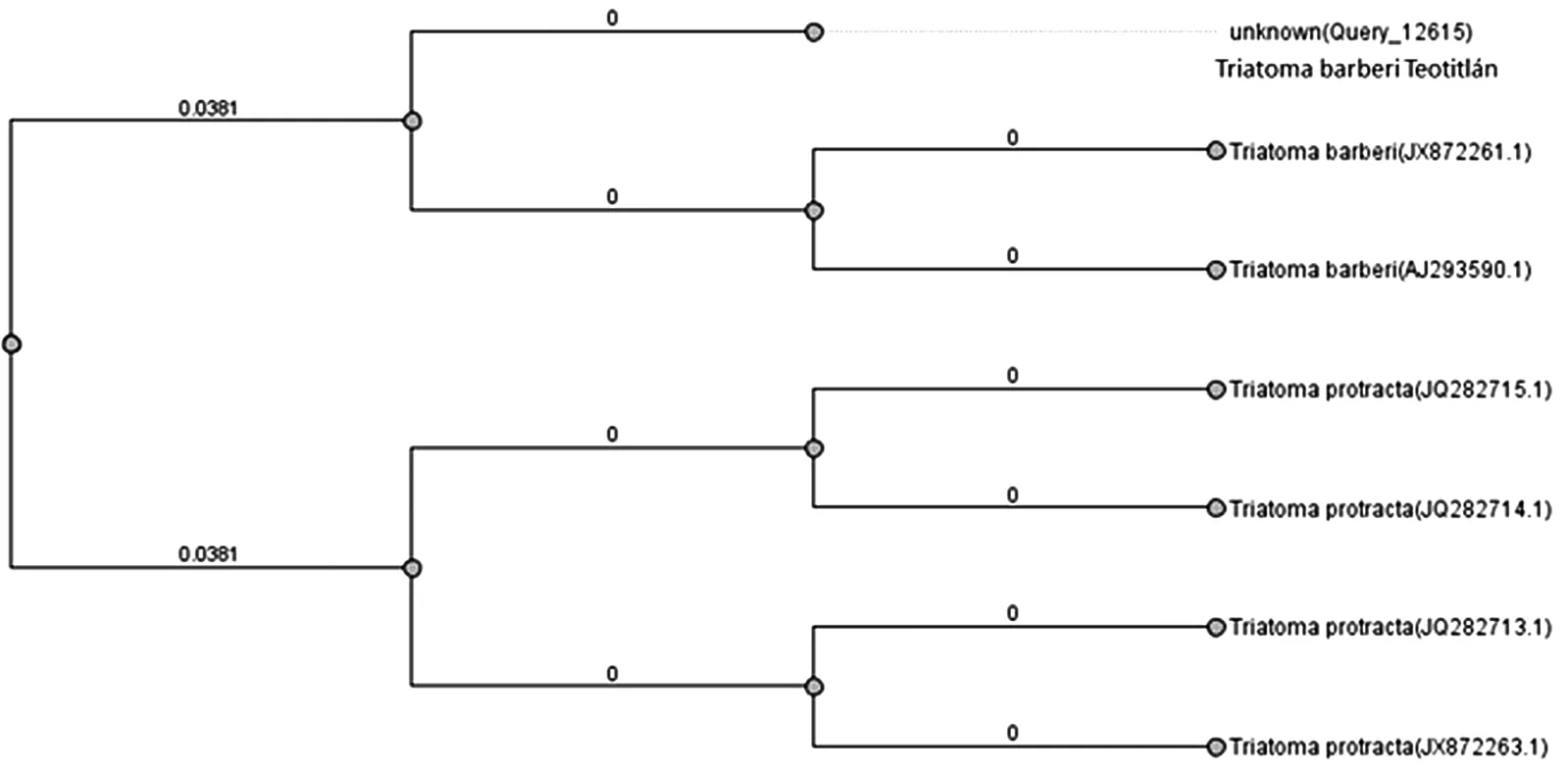
*Triatoma barberi* Neighbour Joining Phylogenetic Tree. *T. barberi* ITS-2 sequence was submitted to the Blastn tool (Query_12615); the Phylogenetic Tree Builder Tool was used to build the phylogenetic tree. The resulting tree shows our specimen belongs to *T. barberi* clade.

Sequences were aligned with the top BLAST hits using the Geneious v4.8.5 software, confirming sequence homology [Supplementary data (Figure A-B, respectively)]. To further determine the identity of the *T. barberi* specimen, a neighbour-joining phylogenetic tree was constructed. Two clearly defined clades were obtained: one for *T. barberi*, including the specimen sequence, and another containing *T. protracta* sequences (Fig. 4).

## DISCUSSION

Vector-borne transmission is the primary route of infection for CD in endemic regions such as Oaxaca. Because the risk of acquiring CD depends on human contact with the infected vector,^15^ ^43^ identifying the distribution of the vector is essential for effective prevention and control strategies.^44^

Triatomines collected in Teotitlán District were identified using entomological and molecular methods. Morphological identifications followed the criteria previously reported by Lent and Wygodzinsky.^26^ Molecular confirmation was then performed using the ITS-2 sequencing. Both approaches confirmed that the MVF specimen was *T. dimidiata*. Haplogroup identification performed by spot pattern analysis^27^ assigned the specimen within haplogroup H2. This finding was consistent with previous reports indicating that *T. dimidiata* H2 is restricted to Mexico, extending from the Isthmus region of Oaxaca to Veracruz and Tabasco,^45^ Yucatan Peninsula, and all Pacific populations north of the Isthmus of Tehuantepec.^46^ Notably, our results extend the known distribution of this haplogroup to northern Oaxaca. In addition, phylogenetic analysis using the *T. barberi* sequence demonstrated that our specimen belongs to the *T. barberi* clade.^47^

Although *T. barberi* and *T. dimidiata* have been reported in Oaxaca,^16^ ^25^ we report the presence of both species in Teotitlán District for the first time [Fig. 1, Supplementary data (Table II)]. Because triatomines have limited mobility, long distance dispersal generally occurs passively and is often associated with human migration from endemic to non-endemic areas.^48^ However, it remains unclear whether the presence of *T. dimidiata* and *T. barberi* in the municipalities of Teotitlán District resulted from passive dispersal^1^ or their displacement from surrounding sylvatic habitats, as these vectors can disperse up to 60 m from such habitats and are strongly attracted to houses.^49^

*Triatoma dimidiata* is widely distributed in Mexico and is considered one of the primary vectors for the transmission of CD in domestic^16^ and peridomestic habitats, although the sylvatic populations can also contribute to *T. cruzi* transmission to humans.^22^ The MVF municipality appears to provide ideal conditions for the range expansion of *T. dimidiata* between sylvatic, peridomestic, and domestic habitats, facilitating access to different reservoir hosts and increasing the risk of *T. cruzi* transmission.^19^ This risk is further enhanced by the species’ strong capacity for domestic adaptation.^1^ ^24^ *T. dimidiata* has been reported at altitudes up to 1287 masl,^37^ consistent with its presence in this municipality (altitude 1,140 masl). Optimal temperatures for vector development range from 16 to 28ºC.^50^ Therefore, the relatively low temperatures in MVF (13ºC), may promote the intradomicile colonisation by the vector. Similarly, the environmental conditions in the TFM municipality are compatible with those previously reported as optimal for *T. barberi*,^51^ and the domestic and peridomestic locations where vectors were captured are consistent with the species’ habitat preferences.^18^

In both cases, the expansion of the geographical range and the domiciliation of *T. dimidiata* and *T. barberi* increase the risk of *T. cruzi* transmission to humans.^17^ Although *T. barberi* is generally regarded as a secondary species for CD transmission based on its ability to colonise artificial structures,^18^ *T. dimidiata* is recognised as a major contributor to CD epidemiology.^18^ ^22^ Despite reports indicating that the H2 haplogroup of *T. dimidiata* has a relatively low *T. cruzi* infection rate,^45^ these findings suggest that *T. dimidiata*’s range expansion has affected CD epidemiology, and that infection with *T. cruzi* in the H2 haplogroup might depend on the presence of infected hosts in this region. The present study originated from a broader serological survey conducted in the study region, during which triatomine specimens were opportunistically collected through community participation. These results demonstrate the potential risks posed by triatomine infestations to *T. cruzi* infection and CD epidemiology.

Although Mexico is one of the six countries (Brazil, Chile, Ecuador, Mexico, Paraguay, and Uruguay) with established regulations to report acute and chronic cases for epidemiological purposes,^7^ access to diagnosis is limited.^15^ This limitation is associated with insufficient public health policies and inadequate awareness of vector distribution and CD transmission risks.^7^ As a result of the lack of free testing services, many infected individuals remain undiagnosed and untreated.^15^ Limited awareness of CD and its vectors within a given community contributes to the spread of CD into non-endemic areas, increasing morbidity and mortality.^52^ Because CD epidemiology is closely linked to vector distribution, interruption of the vectorial transmission remains the most effective control strategy.^20^ By 2024, interruption of domiciliary transmission had been verified nationally in Chile, Paraguay, and Uruguay, and subnationally in Argentina, Brazil, Colombia, Peru, and Bolivia.^7^ Increasing awareness of vector presence among public health and government authorities regarding the presence and redistribution of triatomine vectors could support the establishment of effective entomological surveillance and vector control programmes, thereby reducing the risk of future CD outbreaks. In conclusion, monitoring the redistribution of triatomine species is critical for preventing the spread of CD into non-endemic regions of Mexico and for identifying emerging endemic zones for epidemiological surveillance. These findings highlight the value of community participation as a complementary tool for entomological surveillance.

## SUPPLEMENTARY MATERIALS

Supplementary material

## Data Availability

The contents underlying the research text are included in the manuscript.
