# Supplementary material for: Range expansion of two Triatominae species in Oaxaca, Mexico: first report and molecular identification of Triatoma barberi and Triatoma dimidiata in the Cañada region
Source: Mem Inst Oswaldo Cruz. 2026 Oct 16;121:e260093. doi: 10.1590/0074-02760260093 (PMC13674106; doi:10.1590/0074-02760260093)
Supplement: Supplementary file 1 [file 1678-8060-mioc-121-e260093-s1.pdf]

TABLE I

Code of municipalities of Oaxaca State where Triatomine species have been reported

Municipality name	Code	Municipality name	Code	Municipality name	Code
Asunción Ixtaltepec	5	Cerro de la Cruz	171	Santa Cruz Itundujia	377
Asunción Nochixtlán	6	Juan Bautista Cuicatlán Coyula	177	Santa Cruz Papalutla	380
Asunción Tlacolulita	8	San Juan Bautista Guelache	178	Santa Cruz Zenzontepec	386
El Barrio de la Soledad	10	San Juan Bautista Tlacoatzintepec	182	Santa Gertrudis	387
Calihuala	11	San Juan Bautista Tlachichilco	183	Santa María Apazco	395
Los Horcones	12	San Juan Bautista Tuxtepec	184	Heroica Ciudad de Tlaxiaco	397
Ciudad Ixtepec	14	San Juan Comaltepec	189	Santa María Comatlán	400
Coicoyán de las Flores	16	San Juan Chilateca	192	Santa María Colotepec	401
La Compañía	17	San Juan Guichicovi	198	Santa María Coyotepec	403
Cosolapa	21	San Juan Juquila Mixes	200	Villa de Chilapa de Díaz	405
Chiquihuitlán de Benito Juárez	27	San Juan Lachao	202	Santa María Chilchotla	406
Fresnillo de Trujano	32	San Juan Ozolotepec	211	Santa María del Rosario	408
Guadalupe de Ramírez	34	San Juan Quiotepec	214	Santa María del Tule	409
Guevea de Humboldt	36	San Juan Teitipac	219	Santa María Ecatepec	410
Heroica Ciudad de Huajuapan de León	39	San Juan Yucuita	224	Santa María Huatulco	413
Ixtlan de Juárez	42	San Lorenzo	225	Santa María Jalapa del Marqués	418
Juchitan	43	San Lorenzo Victoria	230	Santa María Tonameca	439
Magdalena Apasco	45	San Martin Peras	242	Santa María Zacatepec	447
Magdalena Mixtepec	48	San Mateo Cajonos	246	Santa María Zoquiatlán	449
Mariscala de Juárez	55	San Miguel Amatlán	261	Santiago Astata	453
El Gavilan	57	San Miguel del Puerto	266	Santiago Ayuquililla	455
Miahuatlán de Porfirio Díaz	59	San Miguel Quetzaltepec	275	Santiago Camotlán	457
Monjas Miahuatlán	61	Villa Sola de Vega	277	Santiago Chazumba	459
Nazareno Etla	63	Temazcal San Miguel Soyaltepec	278	Santiago Choapam	460
Nejapa de Madero	64	San Miguel Tlacotepec	286	Santiago Jamiltepec	467
Santo Domingo Ingenio	66	San Pablo Huitzo	294	Santiago Jocotepec	468
Oaxaca de Juárez	67	San Pablo Macuiltianguis	296	Santiago Juxtlahuaca	469
La Chilana	72	San Pablo Villa de Mitla	298	Santiago Pinotepa Nacional	482
Putla Villa de Guerrero	73	San Pedro Apóstol	301	Santiago Suchilquitongo	483
Reyes Etla	77	San Pedro Atoyac	302	Santiago Yaveo	498
Rojas de Cuauhtémoc	78	San Pedro Huamelula	307	Santiago Yosondúa	500
Salina Cruz	79	San Pedro Mártir	315	Santo Domingo Ingenio	505
San Agustín Atenango	81	San Pedro Mártir Quiechapa	316	Santo Domingo Chihuitán	508
San Agustín de las Juntas	83	San Pedro Mixtepec, Chila	318	Santo Domingo de Morelos	509
San Agustín Loxicha	85	Pochutla	324	Santo Domingo Petepa	513
San Andrés Lagunas	93	San Pedro Quiatoni	325	Santo Domingo Tehuantepec	515
San Andrés Zautla	102	San Pedro Sochiapam	326	Santo Domingo Tlatayápam	518
San Antonio de la Cal	107	San Pedro Totolapam	333	Santo Domingo Tomaltepec	519
San Baltazar Loxicha	113	Villa de Tututepec de Melchor Ocampo	334	Santo Domingo Yanhuitlan	523
San Bartolo Coyotepec	115	San Pedro Yólox	336	Santos Reyes Nopala	526
San Bartolomé Loxicha	117	Villa de Etla	338	Silacayoápam	537
San Bartolo Yautepec	122	San Pedro Y San Pablo Teposcolula	339	Tataltepec de Valdés	543
San Carlos Yautepec	125	San Sebastian Ixcapa	345	Teotitlán de Flores Magón	545
San Dionisio Ocotepec	131	San Sebastián Tecomaxtlahuaca	348	Teotitlán del Valle	546
Arroyo Seco	134	San Sebastián Tutla	350	Tepelmeme Villa de Morelos	548
San Felipe Tejálapam	135	San Simón Zahuatlán	352	Tlacolula de Matamoros	551
San Sebastián Yutanino	137	Santa Ana Cuauhtémoc	355	Tlalixtac de Cabrera	553
San Francisco Lachigolo	145	Santa Ana Yareni	359	Totontepec Villa de Morelos	554
San Jerónimo Coatlán	159	Santa Catarina Cuixtla	362	Trinidad Zaachila	555
San José Ayuquila	165	Santa Catarina Juquila	364	Villa Díaz Ordaz	560
				Zimatlán de Álvarez	570

TABLE II

Distribution of Triatomine species across municipalities in Oaxaca, Mexico. The distribution of eight Triatomine species in the different municipalities (M) of Oaxaca State [Supplementary data (Table I) is shown]

#Species per municipality	*Triatoma barberi*	*Triatoma dimidiata*	*Triatoma mazzottii*	*Triatoma phyllosoma*	*Triatoma pallidipennis*	*Triatoma bolivari*	*Triatoma nitida*	*Rhodnius prolixus*
5	M-64[1,2]	M-64[1,19]	M-64[1,19]	M-64[1,2,19]				M-64[1]
4		M-401[1]	M-401[1,2]	M-401[2,19]		M-401[2]		
		M-439[19]	M-439[1,2]	M-439[1,2,19]		M-439[1,2]		
3	M-17[1,2]	M-17[1,2,7]	M-17[1]					
		M-43[1]		M-43[1,19]			M-43[1]	
			M-55[1]	M-55[1]	M-55[1,2]			
			M-67[1]		M-67[1]	M-67[10]		
	M-81[1,7]	M-81[19]			M-81[1,2]			
		M-85[1,2]	M-85[1]	M-85[2]				
	M-125[19]		M-125[3,19]	M-125[1,2,19]				
	M-230([1,2]	M-230[1,7]			M-230[1]			
	M-261[1]			M-261[19]	M-261[1,2]			
	M-266[19]	M-266[1]	M-266[1,2,19]					
	M-198[19]	M-198[1,2,7,17,19]	M-198[2]					
		M-307 [1,2,7,17]	M-307[3]	M-307[19]				
		M-318[1,11]	M-318[1,2]	M-318[1,19]				
		M-334[1]	M-334[1,2]		M-334[1]			
			M-453[3]		M-453[19]	M-453[1]		
		M-447[19]	M-447[2]			M-447[1]		
	M-457[19]	M-457[2,3,7,19]		M-457[19]				
		M-467[1,19]	M-467[1,2]	M-467[1,19]				
		M-482[19]	M-482[1,2]	M-482[1,19]				
	M-500[1,2]		M-500[3]	M-500[19]				
2		M-5[19]		M-5[2,19]				
	M-12[1]			M-12[2,19]				
	M-14[19]			M-14[1,2,19]				
		M-27[1,13,16,17,19]		M-27[19]				
	M-57[19]						M-57[2]	
	M-59[1,2,17]	M-59[1]						
		M-66[1]	M-66[3]					
		M-73[1]			M-73[1]			
			M-79[3]	M-79[1,19]				
	M-102[1,7]	M-102[1,2]						
	M-115[1,18]		M-115[3]					
		M-117[19]		M-117[2]				
	M-131[1,2]	M-131[19]						
			M-134[2]	M-134[19]				
	M-145[19]	M-145[1]						
	M-177[1,13,16,18]	M-177[1]						
	M-189[1,7,19]	M-189[1,2]						
	M-275[19]	M-275[1,2,19]						
	M-277[1,13,16,18,19]	M-277[1]						
		M-278[1]						M-278[1]
		M-324[1]	M-324[3]					
	M-348[1]				M-348[1]			
			M-418[1,2]	M-418[19]				
		M-449[1]		M-449[1,2,19]				
		M-460[1,13,16,18]	M-460[2]					
		M-505[1]	M-505[2]					
			M-515[3]	M-515[1,2,19]				
	M-523[1]		M-523[3]					
		M-526[1,12-16]	M-526[2]					
					M-537[1]		M-537[1]	
	M-543[1]					M-543[1]		
	M-545**	M-545**						
	M-551[1,2,19]			M-551[19]				

#Species per municipality	*Triatoma barberi*	*Triatoma dimidiata*	*Triatoma mazzottii*	*Triatoma phyllosoma*	*Triatoma pallidipennis*	*Triatoma bolivari*	*Triatoma nitida*	*Rhodnius prolixus*
	M-6[1]	M-16[1]	M-113[3]	M-8[1,2]	M-32[1]	M-159[1]		
	M-11[1]	M-21[19]	M-171[2]	M-10[1]	M-34[1]			
	M-39[1,7]	M-36[1,2]	M-345[2]	M-122[1]	M-165[1]			
	M-45[1]	M-42[1,19]	M-386[3]	M-325[1,2]	M-355[1]			
	M-48[1,7]	M-137[1,2]	M-406[3]	M-333[1,2,19]	M-359[1]			
	M-61[1]	M-184[19]	M-413[1,2]	M-397[19]				
	M-63[1]	M-200[1,2,7]		M-408[2]				
	M-72[2]	M-202[1,2,7]		M-410[1]				
	M-77[19]	M-214[19]		M-459[1]				
	M-78[1,2]	M-246[2]		M-498[19]				
	M-83[19]	M-326[1,19]		M-518[19]				
	M-93[1]	M-336[19]						
	M-107[19]	M-364[1]						
	M-135[1,18]	M-377[1]						
	M-178[1,2]	M-468[1]						
	M-182[1]	M-513[1]						
	M-183[1,2]	M-554[1,2,7]						
	M-192[1]	M-570[1]						
	M-211[1]							
	M-219[1]							
	M-224[1]							
	M-225[1,2]							
	M-242[1]							
	M-286[1]							
	M-294[1]							
	M-296[1]							
1	M-298[1]							
	M-301[1,2]							
	M-302[1]							
	M-315[1,2]							
	M-316[1,2]							
	M-338[19]							
	M-339[1,19]							
	M-350[1,2]							
	M-352[1,2]							
	M-362[1,2]							
	M-380[1,2,7]							
	M-387[2]							
	M-395[1,7,16,18]							
	M-400[1,7]							
	M-403[1,2,19]							
	M-405[1]							
	M-409[19]							
	M-455[1]							
	M-469[1]							
	M-483[1,2]							
	M-508[1,2]							
	M-509[1,2]							
	M-519[1,17]							
	M-546[1,2,7]							
	M-548[1,13,16,18]							
	M-553[19]							
	M-560[1,2]							

**Reported in this work.

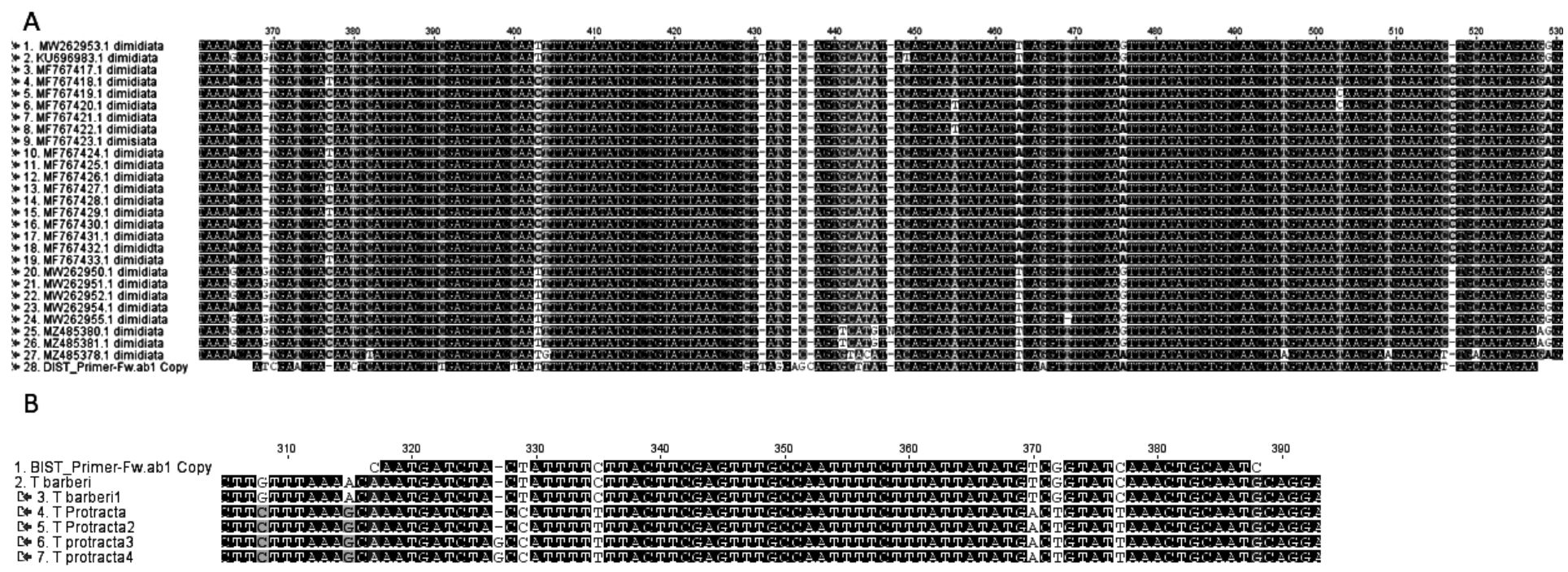

Sequence alignment of the Internal Transcribed Spacer-2 (ITS-2) region confirming species identity by BLAST comparison. Alignments were generated in Geneious v4.8.5. (A) Alignment of the *Triatoma dimidiata* query sequence (DIST_Primer-Fw.ab1) with the top BLAST hits retrieved from GenBank (accession numbers shown), confirming homology with *T. dimidiata* reference sequences. (B) Alignment of the *Triatoma barberi* query sequence (BIST_Primer-Fw.ab1) with *T. barberi* and *Triatoma protracta* reference sequences, confirming homology with the *T. barberi* clade.

REFERENCES

1. GBIF Secretariat. *Triatoma barberi* Usinger, 1939. GBIF Backbone Taxonomy. 2019. Available from: https://doi.org/10.15468/39omei.
2. Ramsey JM, Ordoñez R, Cruz-Celis A, Alvear A, Chávez VJ, López R, et al. Distribution of domestic triatominae and stratification of Chagas Disease transmission in Oaxaca, Mexico. Med Vet Entomol. 2000; 14(1): 19-30.
3. GBIF Secretariat. *Triatoma dimidiata* (Latreille, 1811). GBIF Backbone Taxonomy. 2019. Available from: https://doi.org/10.15468/39omei.
4. GBIF Secretariat. *Triatoma mazzottii* Usinger, 1941. GBIF Backbone Taxonomy. 2019. Available from: https://doi.org/10.15468/39omei.
5. GBIF Secretariat. *Triatoma phyllosoma* (Burmeister, 1835). GBIF Backbone Taxonomy. 2019.
6. GBIF Secretariat. *Rhodnius prolixus* Stål, 1859. GBIF Backbone Taxonomy. 2019.
7. Vidal-Acosta V, Ibáñez-Bernal S, Martínez-Campos C, editors. Infección natural de chinches Triatominae con *Trypanosoma cruzi* asociadas a la vivienda humana en México. 2000.
8. GBIF Secretariat. *Triatoma nitida* Usinger, 1939. GBIF Backbone Taxonomy. 2019. Available from: https://doi.org/10.15468/39omei.
9. GBIF Secretariat. *Triatoma pallidipennis* (Stål, 1872). GBIF Backbone Taxonomy. 2019. Available from: https://doi.org/10.15468/39omei.
10. GBIF Secretariat. *Triatoma bolivari* Carcavallo, Martínez & Pelaez, 1984. GBIF Backbone Taxonomy. 2019. Available from: https://doi.org/10.15468/39omei.
11. Goldsmith RS, Zárate RJ, Zárate LG, Morales G, Kagan I, Drickey R, et al. Clinical and epidemiologic studies of Chagas' disease in rural communities of Oaxaca, Mexico, and an eight-year followup: II. Chila. Bull Pan Am Health Organ. 1992; 26(1): 47-59.
12. Tay JS, Salazar-Schettino PM, Bucio MI, Zarate R, Zarate L. Estado actual de nuestros conocimientos sobre la enfermedad de Chagas en la República Mexicana. Rev Soc Bras Med Trop. 1981; 14: 29-67.
13. Zárate LG. A checklist of the Triatominae Hemiptera Heteroptera Reduviidae of Mexico. Int J Entomol. 1985; 27(1-2): 102-27.
14. Goldsmith RS, Zárate RJ, Zárate LG, Kagan I, Jacobson LB. Clinical and epidemiologic studies of Chagas' disease in rural communities in Oaxaca State, Mexico, and a seven-year follow-up: I. Cerro del Aire. Bull Pan Am Health Organ. 1985; 19(2): 120-38.
15. Ramirez G, Mas J, Ramsey J. Patrones espaciales asociados a la infestación comunitaria por vectores de la enfermedad de Chagas. Rev Cartogr. 2020: 41-59.
16. Salazar-Schettino PM, Bucio-Torres MI, de Haro-Arteaga I. Reservorios y transmisores de *Trypanosoma cruzi* en el estado de Oaxaca. 1987. 1987; 29(1): 7.
17. Salazar-Schettino P, De Haro-Arteaga I, Cabrera-Bravo M. Tres especies de triatominos y su importancia como vectores de *Trypanosoma cruzi* en México. Medicina (B Aires). 2005; 65(1): 63-9.
18. Hernández R, Herrera J, Bosseno MF, Brenière SF, Espinoza B. *Trypanosoma cruzi*: data supporting clonality in Mexican stocks. J Parasitol. 2001; 87(5): 1178-81.
19. Fernández-Santos NA, Trujillo-García JC, Hamer SA, Wei L, Martínez-Montoya H, Tamez-Guerra P, et al. Domestic *Triatoma* spp. infections with *Trypanosoma cruzi*, household infestations, and molecular identification in Oaxaca, México. Insects. 2022; 13(12): 1134.
