# Supplementary material for: Range expansion of two Triatominae species in Oaxaca, Mexico: first report and molecular identification of Triatoma barberi and Triatoma dimidiata in the Cañada region
Source: Mem Inst Oswaldo Cruz. 2026 Oct 16;121:e260093. doi: 10.1590/0074-02760260093 (PMC13674106; doi:10.1590/0074-02760260093)
Supplement: Peer review history

# Reviewer report: FIRST REVIEW ROUND - REVIEWERS’ COMMENTS

## REVIEWER #1

This study represents a large surveillance program to document different triatomine species found in Oaxaca Mexico. The specimens were identified morphologically and molecularly. Several new species records are being documented for different parts of Oaxaca. Overall this is an important body of work. Below I am documenting some specific areas of the manuscript that could use improvement.

Page 4. Ln. 24. Is ‘morphometric’ a better term than ‘quantitative Imagery Identification’?

Page 5. Ln. 36-40. Please clarify which time period this refers to. It says ‘by the end of 2025’ but does that mean ‘in the year 2025’? And are those 73 cases in Oaxaca all chronic?

Page 5. Ln. 49. I recently heard there might be members of the triatominae subfamily that don’t feed on blood. To keep this statement accurate, this modification could be made: “and all hematophagous species are capable of T. cruzi transmission.”

Page 6. Ln. 8-13. “The transmission of T. cruzi depends not only on the type, number, distribution, and feeding habits of vector species in domestic, peridomestic, or sylvatic habitats, but also on environmental and host factors [17, 18].” In this sentence it is hard to understand what you mean by ‘environmental and host factors’.

Page 7 Ln. 33. Italicize T. dimidiata.

Page 9 Ln. 24. The abbreviation ‘MVF’ is used frequently but is there a location that defines what this means? Same for TFM.

Page 11. Ln. 24. ‘the absence of reports citing the presence’ could be changed to ‘no documented occurrences’.

Page 15. Ln. 50. The eyes were white. If this is referring to the specimen in Fig 3B, I assume that is because this individual was found dead and desiccated? The broken legs and antennae would also suggest it was dead and desiccated.

Page 16, Ln 33. You don’t need the “’s” at the end of T. barberi’s.

Page 20. Ln. 52. This statement is inaccurate. Please read the WHO report again and you will see your error. Not the entire country has had interrupted domiciliary transmission for five of them like you are claiming. Below is the quoted section from the WHO report.

“As of December 2024, interruption of domiciliary vectorial transmission had been verified in the entire national territories of three countries in the Region of the Americas: Chile, Paraguay and Uruguay, and in a proportion of the national territories of five other countries: Argentina, Brazil, Colombia, Peru and the Plurinational State of Bolivia, for a total of eight countries.”

Figure 2. I don’t see what you mean by “Newly reported occurrences of T. barberi and T. dimidiate are highlighted in the insert (TFM and MVF).”

## REVIEWER #2

Thank for submitting this important work in the State of Oaxaca. This will lead to ongoing discussion of vector control and awareness of vector-borne transmission risks for Chagas disease in this region of southern Mexico. I believe the manuscript is well written but needs some additional information and I have some suggestions to amplify the importance of your work.

Comments and Suggestions:

Abstract:

Please update by indicating the United States is in endemic for Chagas disease. “CD is endemic in 21 Latin American countries and the United States,....”

Please include dates of investigation in Methods portion of abstract.

Introduction:

Please also include the United States as an endemic country for CD. Reference 1 below. Please also update some of your global epidemiological data from most recent global assessment of Chagas. Reference 2. below.

Please also include prevalence data for Mexico and Oaxaca from reference 3. below. This publication has additional data.

I also suggest that you include in the Introduction the potential for under-reporting of Chagas disease in Mexico and in Oaxaca specifically.

At the end of your Introduction I like the statements on One Health and targeting a introduction statement about Oaxaca But I recommend adding a statement of the aims of your investigation and hypothesis. This grabs the attention of the readers and also why you did this investigation.

Methods:

Triatomine Collection. This subsection needs attention. Please provide information on the dates of collection, entomological techniques, how you identified regions, program for community scientists, how the bugs were preserved after collecting. Please review this section and improve.

Please provide details on GIS mapping.

Results:

Overall excellent. Map is amazing. I do have a concern. Only 1 triatomine was positive for T. cruzi? That is likely a very unusual finding. It sounds like there was a problem with DNA extraction or preservation of the insect int he field?

Among 130> triatomines you should expect 20-40% or even higher for harboring T. cruzi. I suggestion that this portion of the manuscript be removed and re-evaluated by the authors. Are these bugs stored for further analysis?

In my opinion, the T. cruzi investigation has errors and prevents this manuscript from being published. However, reporting the triatomine data and distribution has importance.

Discussion.

I would limitations to the study. I would also consider removing T. cruzi data and seroprevelance data or statement as this is not published. It adds confusion to what you presenting.

In my opinion this is an excellent entomological descriptive study and shoudl focus on those highlights.

Please add more data on new findings of triatomines in regions that have now identified them in homes and ongoing concerns for Chagas. Example reference: 4. and 5. below.

References to include:

1. Beatty, N. L., Hamer, G. L., Moreno-Peniche, B., Mayes, B., & Hamer, S. A. (2025). Chagas Disease, an Endemic Disease in the United States. Emerging infectious diseases, 31(9), 1691–1697.

2. GBD 2023 Chagas Disease and RAISE Study Collaborators (2026). Global, regional, and national burden of Chagas disease, 1990-2023: a systematic analysis for the Global Burden of Disease Study 2023. The Lancet. Infectious diseases, 26(3), 284–301.

3. Arnal, A., Waleckx, E., Rico-Chávez, O., Herrera, C., & Dumonteil, E. (2019). Estimating the current burden of Chagas disease in Mexico: A systematic review and meta-analysis of epidemiological surveys from 2006 to 2017. PLoS neglected tropical diseases, 13(4), e0006859.

4. Fernández-Santos, N. A., Trujillo-García, J. C., Hamer, S. A., Wei, L., Martínez-Montoya, H., Tamez-Guerra, P., Hamer, G. L., & Rodríguez-Pérez, M. A. (2022). Domestic Triatoma spp. Infections with Trypanosoma cruzi, Household Infestations, and Molecular Identification in Oaxaca, México. Insects, 13(12), 1134.

5. Beatty, N. L., Bhosale, C. R., White, Z. S., Torhorst, C. W., Wilson, K. N., Dorleans, R., Stenn, T. M. S., Killets, K. C., Curtis-Robles, R., Burkett-Cadena, N., Nováková, E., Hamer, G. L., Hamer, S. A., & Wisely, S. M. (2025). Field evidence of Trypanosoma cruzi infection, diverse host use and invasion of human dwellings by the Chagas disease vector in Florida, USA. PLoS neglected tropical diseases, 19(7), e0012920.

Overall, this manuscript is publishable but will need major revision. This includes removal of T. cruzi investigation among the triatomines as this appears to have errors in methods of detection.

## AUTHORS’ RESPONSE TO THE REVIEWERS

Dear Dr. Ademir de Jesus Martins Jr.,

Handling Editor

Memórias do Instituto Oswaldo Cruz

RE: Revised Manuscript ID: MIOC-2026-0093

We thank you and the reviewers for the careful evaluation of our manuscript entitled “Range Expansion of Two Triatominae Species in Oaxaca, Mexico: First Report and Molecular Identification of Triatoma barberi and Triatoma dimidiata in the Cañada Region” (Manuscript ID: MIOC-2026-0093).

We greatly appreciate the constructive comments and suggestions provided during the review process. We have carefully considered all recommendations and revised the manuscript accordingly. In particular, we addressed the concerns regarding the reliability and interpretation of the Trypanosoma cruzi infection data by removing this component from the manuscript and refocusing the study on its principal contribution: the entomological and molecular identification of Triatoma species and their geographic distribution in the Cañada region of Oaxaca.

Please find attached a detailed point-by-point response to all reviewer comments. Our responses are provided directly below each reviewer comment, which has been reproduced in bold for clarity. All modifications introduced in the revised manuscript are identified by page and line numbers to facilitate evaluation.

We sincerely appreciate the opportunity to revise this work and hope that the revisions adequately address the concerns raised by the reviewers and editors. We trust that the manuscript, in its revised form, will be suitable for publication in Memórias do Instituto Oswaldo Cruz.

Sincerely,

Dr. Patricia González-Cano

On behalf of all authors

REVIEWER COMMENTS AND THE RESPONSES:

We thank reviewers 1 and 2 for their comments and suggestions, which are presented in bold below. Please also find our responses to each comment, along with a summary of the corresponding revisions made to address the specific points raised.

## Reviewer 1:

Page 8. Ln. 21. Is ‘morphometric’ a better terminology than ‘quantitative Imagery Identification’?

Response: The phrase “quantitative imagery identification” has been replaced with “image-based dorsal spot pattern analysis” to more accurately reflect the methodological approach used for species identification.

Page 4. Ln. 19-21. Please clarify which time period this refers to. It says, ‘by the end of 2025’, but does that mean ‘in the year 2025’? And are those 73 cases in Oaxaca all chronic?

Response: We have revised the text to clearly indicate that the data refers to cumulative cases reported up to the end of 2025. We also clarified the distribution of cases in Oaxaca state, specifying that of the 78 cases reported, 5 were acute and 73 were chronic.

Page 5. Ln. 2-3. I recently heard there might be members of the Triatominae subfamily that don’t feed on blood. To keep this statement accurate, this modification could be made: “and all hematophagous species are capable of *T. cruzi* transmission.”

Response: We have revised the sentence accordingly to specify that all hematophagous species are capable of *T. cruzi* transmission. This modification improves the accuracy of the statement.

Page 5. Ln. 10-13. “The transmission of *T. cruzi* depends not only on the type, number, distribution, and feeding habits of vector species in domestic, peridomestic, or sylvatic habitats, but also on environmental and host factors [17, 18].” In this sentence it is hard to understand what you mean by ‘environmental and host factors.’

Response: We have revised the sentence to clarify the meaning of environmental and host factors. Specifically, we now indicate that these factors include host availability and environmental conditions influencing feeding behavior, as some Triatomine species feed on specific hosts depending on their presence in domestic or peridomestic habitats or opportunistically on mammals in sylvatic environments.

Page 8 Ln. 2. Italicize *T. dimidiata*.

Response: The species name, *T. dimidiata,* has been italicized throughout the text as suggested.

Page 7 Ln. 23; Page 8 Ln. 1. The abbreviation ‘MVF’ is used frequently but is there a location that defines what this means? Same for TFM.

Response: The abbreviations, TFM and MVF, representing Teotitlán de Flores Magón and Mazatlán Villa de Flores respectively, have now been defined at their first occurrence in each relevant section of the manuscript.

Page 12. Ln. 12. ‘the absence of reports citing the presence’ could be changed to ‘no documented occurrences.’

Response: We thank the reviewer for this suggestion. The sentence has been revised accordingly to use “no confirmed occurrences,” improving clarity and readability.

Page 14. Ln. 22. The eyes were white. If this is referring to the specimen in Fig 3B, I assume that is because this individual was found dead and desiccated? The broken legs and antennae would also suggest it was dead and desiccated.

Response: We thank the reviewer for this observation. We have revised the description of the specimen to correct the eye color. The eyes are now described as black, which accurately reflects the specimen examined. The corresponding text has been updated accordingly.

Page 15, Ln 18. You don’t need the “’s” at the end of *T. barberi’s*.

Response: The possessive form has been corrected accordingly.

Page 19. Ln. 17-22. This statement is inaccurate. Please read the WHO report again and you will see your error. Not the entire country has had interrupted domiciliary transmission for five of them like you are claiming. Below is the quoted section from the WHO report.

Response: We have revised the statement according to the WHO report. The manuscript now specifies that, by 2024, interruption of domiciliary transmission was verified at national level in Chile, Paraguay, and Uruguay, and at subnational level in Argentina, Brazil, Colombia, Peru, and Bolivia. “As of December 2024, interruption of domiciliary vectorial transmission was verified in the entire national territories of three countries in the Region of the Americas: Chile, Paraguay and Uruguay, and in a proportion of the national territories of five other countries: Argentina, Brazil, Colombia, Peru and the Plurinational State of Bolivia, for a total of eight countries.”

Figure 2. I don’t see what you mean by “Newly reported occurrences of *T. barberi* and T. dimidiate are highlighted in the insert (TFM and MVF).”

Response: We have revised Figure 2 and its legend for clarity. The inset has been updated to clearly indicate the locations of Teotitlán de Flores Magón (TFM, panel A) and Mazatlán Villa de Flores (MVF, panel B), where the newly reported occurrences were recorded.

## Reviewer: 2

Abstract:

Please update by indicating the United States is in endemic for Chagas disease. “CD is endemic in 21 Latin American countries and the United States,”

Response Page 3 Ln 4: We thank the reviewer for this valuable suggestion. The Introduction has been revised accordingly to include the United States as an endemic region for Chagas disease. The wording has also been slightly refined to improve clarity.

Please include dates of investigation in Methods portion of abstract.

Response Page 3. Ln. 13: We thank the reviewer for this suggestion. The Methods section of the Abstract has been revised to include the study period. Specifically, we now indicate that the literature review and data collection were conducted between 2019 and 2024.

Introduction:

Please also include the United States as an endemic country for CD. Reference 1 below.

Response Page 4. Ln. 13: The Introduction has been revised to include the United States of America as an endemic region for Chagas disease, and the suggested reference has been incorporated in the reference list.

Please also update some of your global epidemiological data from most recent global assessment of Chagas. Reference 2. below.

Response Page 4. Ln. 9- 13 The epidemiology has been updated and the suggested reference included.

Please also include prevalence data for Mexico and Oaxaca from reference 3. below. This publication has additional data.

Response Page 4 Ln. 9-15: The Introduction has been revised accordingly to incorporate updated global and national epidemiological data on Chagas disease, and the suggested reference has been included. The text has also been refined for clarity and the map and supplementary table 2 updated.

I also suggest that you include in the Introduction the potential for under-reporting of Chagas disease in Mexico and in Oaxaca specifically.

Response: We thank the reviewer for this valuable suggestion. The Introduction has been revised to include a discussion of the potential under-reporting of Chagas disease in Mexico, particularly in high-burden regions such as Oaxaca. This addition highlights the challenges in surveillance and diagnosis and strengthens the context and relevance of our study.

At the end of your Introduction, I like the statements on One Health and targeting an introduction statement about Oaxaca But I recommend adding a statement of the aims of your investigation and hypothesis. This grabs the attention of the readers and why you did this investigation.

Response: The Introduction has been revised to include a clear statement of the study aims and hypothesis, emphasizing the relevance of our investigation within a One Health framework.

Methods:

Triatomine Collection. This subsection needs attention. Please provide information on the dates of collection, entomological techniques, how you identified regions, program for community scientists, how the bugs were preserved after collecting. Please review this section and improve.

Response: We thank the reviewer for this important comment. We would like to clarify that triatomine collection was not the primary objective of this study, which was conducted as part of a seroprevalence survey in the La Cañada region of Teotitlán de Flores Magón, Oaxaca. Specimens were obtained through community participation following educational outreach activities on Chagas disease and its vectors. As a result, collections were opportunistic rather than based on standardized entomological sampling protocols. We have revised the Methods section to clarify the context of specimen acquisition, including community involvement and handling of collected specimens, to improve transparency and reproducibility.

Please provide details on GIS mapping.

Response: We thank the reviewer for this suggestion. The Methods section has been revised to include additional details regarding the geographic data. Geographic coordinates of collection localities were recorded and used exclusively for descriptive mapping purposes. No spatial statistical analyses were performed.

Results:

Overall excellent. Map is amazing. I do have a concern, only 1 triatomine was positive for *T. cruzi*? That is likely a very unusual finding. It sounds like there was a problem with DNA extraction or preservation of the insect in the field? Among 130> triatomines you should expect 20-40% or even higher for harboring *T. cruzi*.

I suggest that this portion of the manuscript be removed and re-evaluated by the authors. Are these bugs stored for further analysis? In my opinion, the *T. cruzi* investigation has errors and prevents this manuscript from being published. However, reporting the triatomine data and distribution has importance.

Response: We have removed the section related to *Trypanosoma cruzi* detection from the manuscript as suggested. Given the limitations in sample availability and the inability to perform additional confirmatory analyses, we agree that inclusion of these results could lead to uncertainty in their interpretation. The manuscript now focuses on the distribution and occurrence of triatomine vectors, which represents the primary strength of this study.

Discussion.

I would also consider removing *T. cruzi* data and seroprevalence data or statements as this is not published. It adds confusion to what you are presenting.

Response: We thank the reviewer for this important comment. We agree that inclusion of unpublished *T. cruzi* infection data may introduce confusion; therefore, these data have been removed from the revised manuscript. We have retained a brief reference to the seroprevalence component to provide context, as this study originated from a broader serological survey conducted in the La Cañada region of Teotitlán de Flores Magón, Oaxaca. The seroprevalence data are being prepared for publication in a separate manuscript.

Please add more data on new findings of triatomines in regions that have now identified them in homes and ongoing concerns for Chagas. Example reference: 4. and 5. below.

Response: The manuscript has been revised to incorporate additional information on the occurrence of triatomine vectors in domestic environments and their implications for ongoing Chagas disease transmission risk. Specifically, we have updated the distribution maps, tables, and Results section to highlight newly documented occurrences in intradomicile and peridomicile settings. The suggested references have been incorporated, and the ecological and epidemiological relevance of these findings has been expanded.

Overall, this manuscript is publishable but will need major revision. This includes removal of *T. cruzi* investigation among the triatomines as this appears to have errors in methods of detection.

Response: We also acknowledge the concern raised, after careful consideration of, we decided to remove the *T. cruzi* infection component from the manuscript and refocus the study on the entomological and molecular identification findings, which constitute the principal contribution of this work.

# Reviewer report: REVIEWERS’ COMMENTS

## REVIEWER #1

Thank you for providing a revision to the submitted manuscript. Point by point response to Reviewers was sufficient. I agree with the changes made. There is substantial clarity to the findings. This work will be an value to the medical and entomological literature. It will raise awareness for triatomine vector expansion and distribution in Oaxaca. I recommend accept at this time.

## REVIEWER #2

No comments.
